# A plant MinD homologue rescues *Escherichia coli *HL1 mutant (Δ*MinDE*) in the absence of MinE

**DOI:** 10.1186/1471-2180-9-101

**Published:** 2009-05-20

**Authors:** Min Zhang, Yong Hu, Jingjing Jia, Hongbo Gao, Yikun He

**Affiliations:** 1 College of Life Science, Capital Normal University, Beijing 100037, PR China; 2 College of Biological Sciences and Biotechnology, Beijing Forestry University, Beijing 10083, PR China

## Abstract

**Background:**

In *E. coli*, the Min operon (*MinCDE*) plays a key role in determining the site of cell division. MinE oscillates from the middle to one pole or another to drive the MinCD complex to the end of the cell. The MinCD complex prevents FtsZ ring formation and the subsequent cell division at cell ends. In *Arabidopsis thaliana*, a homologue of MinD has been shown to be involved in the positioning of chloroplast division site.

**Results:**

To learn whether the MinD homologue in plants is functional in bacteria, AtMinD was expressed in *E. coli*. Surprisingly, AtMinD can rescue the minicell phenotype of *E. coli *HL1 mutant (Δ*MinDE*) in the absence of EcMinE. This rescue requires EcMinC. AtMinD was localized to puncta at the poles of *E. coli *cells and puncta in chloroplasts without oscillation. AtMinD expressed in the HL1 mutant can cause a punctate localization pattern of GFP-EcMinC at cell ends. Yeast two hybrid and BiFC analysis showed that AtMinD can interact with EcMinC.

**Conclusion:**

Similar to the MinD in *Bacillus subtilis*, AtMinD is localized to the polar region in *E. coli *and interacts with EcMinC to confine EcFtsZ polymerization and cell division at the midpoint of the cell.

## Background

In *Escherichia coli*, proper positioning of the cell division apparatus at midpoint of the cell is mainly controlled by *Min *operon, which encodes MinC, MinD and MinE [1]. FtsZ, a bacteria-type cytoskeleton, self-polymerizes, marks the division site of the cell and recruits other components of the cell division apparatus [2,3]. MinD, a membrane-bound ATPase, recruits MinC to inhibit FtsZ polymerization at the non-division site [4,5]. MinE forms a dynamic ring that undergoes a repetitive cycle of movement first to one pole and then to the opposite pole in the cell [6], and induces conformational changes in membrane-bound MinD [7], which results in release of MinC and conversion of membrane-bound MinD (MinD:ATP) to cytoplasmic MinD (MinD:ADP) [7]. This highly dynamic localization cycle of Min proteins inhibits FtsZ ring formation near cell ends and forces FtsZ and many other cell division proteins to assembly at the center of the cell [8]. FtsZ and Min proteins are conserved in a wide variety of bacteria, including cyanobacteria [9].

As endosymbionts in plant cells, chloroplasts have inherited many characters from their ancestor, cyanobacteria [10]. For example, FtsZ, MinD, MinE and ARC6 are chloroplast division proteins evolved from cyanobacteria cell division proteins [9]. Besides the similarity shared with their ancestors, some new characters were gained in these proteins during evolution. The FtsZ family in *Arabidopsis *includes AtFtsZ1, which lacks the conserved C-terminal domain [11]; AtFtsZ2-1 and AtFtsZ2-2 [12], which are more similar to the FtsZ in cyanobacteria than other members [13]; and ARC3, which has a much less conserved GTPase domain of FtsZ and a later acquired C-terminal MORN repeat domain [14]. All these FtsZ homologues can form a ring at the chloroplast division site [15,16]. Similar to their homologues in bacteria, MinD and MinE in *Arabidopsis *have been shown to be involved in the positioning of the division site in chloroplasts [17-19]. Antisense suppression of *AtMinD *or a single mutation in *AtMinD *cause misplacement of the chloroplast division site in *Arabidopsis *[17,20]. AtMinE antagonizes the function of AtMinD [19]. Overexpression of *AtMinE *in *Arabidopsis *results in a phenotype similar to that caused by antisense suppression of *AtMinD *[19]. However, AtMinD has been shown to be localized to puncta in chloroplasts [20] and never been reported to oscillate. This is quite different from that of EcMinD in *E. coli*.

To study the function of AtMinD, we expressed it in *E. coli *HL1 mutant which has a deletion of *EcMinD *and *EcMinE *and a minicell phenotype [21]. Surprisingly, the mutant phenotype was complemented. Similar to the localization in chloroplasts [20], AtMinD was localized to puncta at the poles in *E. coli *HL1 mutant without oscillation in the absence of EcMinE. We also confirmed that AtMinD can interact with EcMinC. AtMinD may function through EcMinC by prevent FtsZ polymerization at the polar regions of the cell. Our data suggest that the cell division of *E. coli *can occur at the midcell with a non-oscillating Min system which includes AtMinD and EcMinC and the working mechanism of AtMinD in chloroplasts may be different from that of EcMinD in *E. coli*.

## Results and discussion

### A MinD homologue from *Arabidopsis *complements the minicell mutant phenotype of *E. coli *HL1 mutant (ΔMinDE) in the absence of MinE

**Figure 1:**
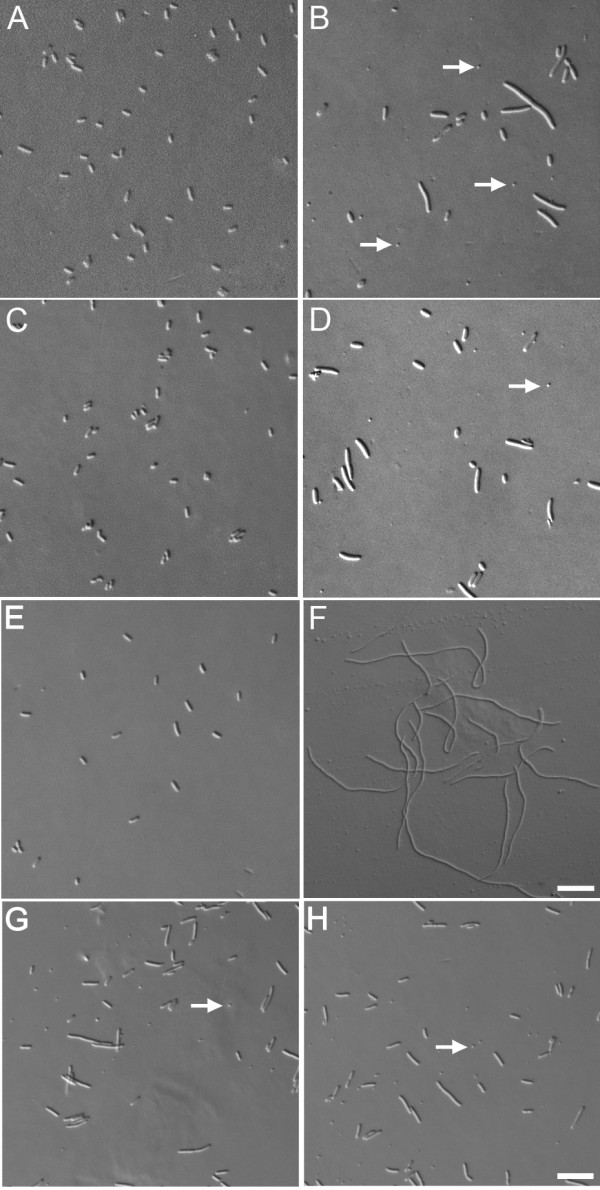
**The phenotype of *E. coli *cells**. (A) Wildtype, DH5α. (B) HL1 mutant (Δ*MinDE*). (C) HL1 mutant (Δ*MinDE*) complemented by *pM1113-MinDE *at 20 μM IPTG. (D) HL1 mutant (Δ*MinDE*) cannot be complemented by *pM1113-AtMinD *at 0 μM IPTG. (E) HL1 mutant (Δ*MinDE*) complemented by *pM1113-AtMinD *at 50 μM IPTG. (F) HL1 mutant (Δ*MinDE*) containing *pM1113-MinD *at 20 μM IPTG. (G) RC1 mutant (Δ*MinCDE*). (H) RC1 mutant (Δ*MinCDE*) containing *pM1113-AtMinD *at 50 μM IPTG. Arrows in (B, D, G and H) mark the minicells. The bar in (A to E, G and H) represents 10 μm; the bar in (F) represents 20 μm.

The *E. coli *HL1 mutant (Δ*MinDE*) has an apparent minicell phenotype with 30.5% of the cells are shorter than 2 μm and 38.1% of the cells are between 2 μm to 5 μm (Figure 1B and Table 1). Actually, most of the cells shorter than 2 μm are minicells that are usually shorter than 1.2 μm. In the wild-type DH5α, only 2.6% of the cells are smaller than 2 μm and 97.4% of the cells are between 2 μm to 5 μm (Figure 1A and Table 1). The mutant phenotype of HL1 mutant was complemented by a *pM1113-MinDE *plasmid with 20 μM IPTG (Figure 1C and Table 1), which was used for the induction of MinD and MinE. Because the homologues of MinD and MinE are involved in the division of chloroplasts in plants [9] and their function may still be conserved, we set up a bacterial system to study their function. Surprisingly, a *pM1113-AtMinD *plasmid can complement the mutant phenotype with 50 μM IPTG in the absence of *EcMinE *or *AtMinE *(Figure 1E, Table 1 and Table 2). We have also grown the *E. coli *HL1 mutant cells (Δ*MinDE*) containing *pM1113-AtMinD *with higher or lower concentration of IPTG, and found the mutant phenotype was recovered best with 50 μM IPTG (Figure 1E and our unpublished results). Minicells were reduced from 30.5% to 8.7% and the cells that are between 2 μm and 5 μm were increased from 38.1% to 87.4% (Table 1). Misplaced septa were also reduced from 55% to 6%, which is close to 3% in DH5α and 1% in the HL1 mutant rescued by EcMinD and EcMinE (Table 2). At higher IPTG concentration, the growth of cells was inhibited and the phenotype was not recovered so well (data not shown). Even without IPTG addition, the mutant phenotype was slightly rescued with a reduction of the cells that were 5–10 μm long from 29% to 5.6% (Table 1). This may be due to a leaky expression of AtMinD. As a control, HL1 mutant cells (Δ*MinDE*) transformed with a *pM1113-EcMinD *plasmid and grown with 20 μM IPTG showed a phenotype of long filaments but not minicells (Figure 1F and Table 1). This indicates that EcMinD is expressed and active but can not complement the mutant phenotype without EcMinE. To further understand the function of AtMinD in *E. coli*, AtMinD was expressed in RC1 mutant (Figure 1G and Table 1) that has a deletion of Min operon, i.e. MinCDE, with 50 μM IPTG. The RC1 mutant has a minicell phenotype similar to that of HL1 mutant. Expression of AtMinD in RC1 mutant couldn't rescue the mutant phenotype. These data suggest that the complementation of HL1 mutant by AtMinD requires the presence of EcMinC.

**Table 1:** Statistical analysis of the cell length

Genotype	IPTG	Minicell (%)	2–5 μm (%)	5–10 μm (%)	>10 μm (%)
DH5α	0 μM	2.6 ± 1.0	97.4 ± 1.0	0	0

HL1	0 μM	30.5 ± 1.0	38.1 ± 2.2	29.0 ± 1.6	2.4 ± 0.3

RC1	0 μM	41.5 ± 3.4	50.4 ± 2.0	7.0 ± 2.4	1.1 ± 0.8

HL1 with EcMinDE	20 μM	0.7 ± 0.3	96.8 ± 0.6	2.3 ± 0.3	0.2 ± 0.0

HL1 with AtMinD	0 μM	40.5 ± 3.1	51.3 ± 3.0	5.6 ± 0.7	2.6 ± 2.3

HL1 with AtMinD	50 μM	8.7 ± 0.8	87.4 ± 2.5	3.9 ± 1.8	0

HL1 with EcMinD	20 μM	0	0	0	100

RC1 with AtMinD	50 μM	31.5 ± 1.5	48.8 ± 1.3	16 ± 4.4	5.5 ± 2.8

HL1 with AtMinD-GFP	50 μM	12.5 ± 2.4	78.6 ± 2.5	7.6 ± 1.1	1.3 ± 0.3

HL1 with GFP-AtMinD	50 μM	5.2 ± 1.5	91.5 ± 2.7	3.3 ± 1.3	0

Shown above are the means ± S.D. obtained from 3 independent repeats. The number of the cells measured in each repeat is between 150 and 200.

**Table 2:** Analysis of the cell division phenotype

Genotype	Cells	Septa	Polar	% Polar	Phenotype
DH5α	867	229	6	3	WT

HL1	991	216	119	55	Min^-^

HL1(P_lac_::*EcMinDE*)	974	232	3	1	WT

HL1(P_lac_::*AtMinD*)	863	161	11	6	WT

HL1(P_lac_::gfp-*AtMinD*)	1081	219	10	5	WT

HL1(P_lac_::*AtMinD-gfp*)	943	137	17	12	WT like

Shown above is the division phenotype analysis of *E. coli *cells with different genotypes. EcMinDE was induced with 20 μM IPTG, AtMinD and its GFP fusion proteins were induced with 50 μM IPTG. Cells: the total number of cell examined; Septa: the total number of septa counted; Polar: the number of septa which were misplaced at or near a cell pole; % Polar: the percentage of septa which were misplaced at or near a cell pole. Min^-^, minicell phenotype. WT, most of the cells have a normal size and no cell or only a small part of the cells are minicells or long filaments.

The sequences of the MinD in bacteria are similar to those in plants [17]. Members of the MinD family have important roles in positioning the FtsZ ring and the division apparatus to either the mid-cell of bacteria or the mid-site of chloroplasts [9]. The complementation of *E. coli *HL1 mutant (Δ*MinDE*) by AtMinD and the requirement of EcMinC for this complementation suggest that the function of MinD is also conserved between bacteria and plants. However, this complementation doesn't require the presence of EcMinE suggests that AtMinD may have some characters different from that of EcMinD.

### AtMinD is localized to puncta in *E. coli *and chloroplasts

**Figure 2:**
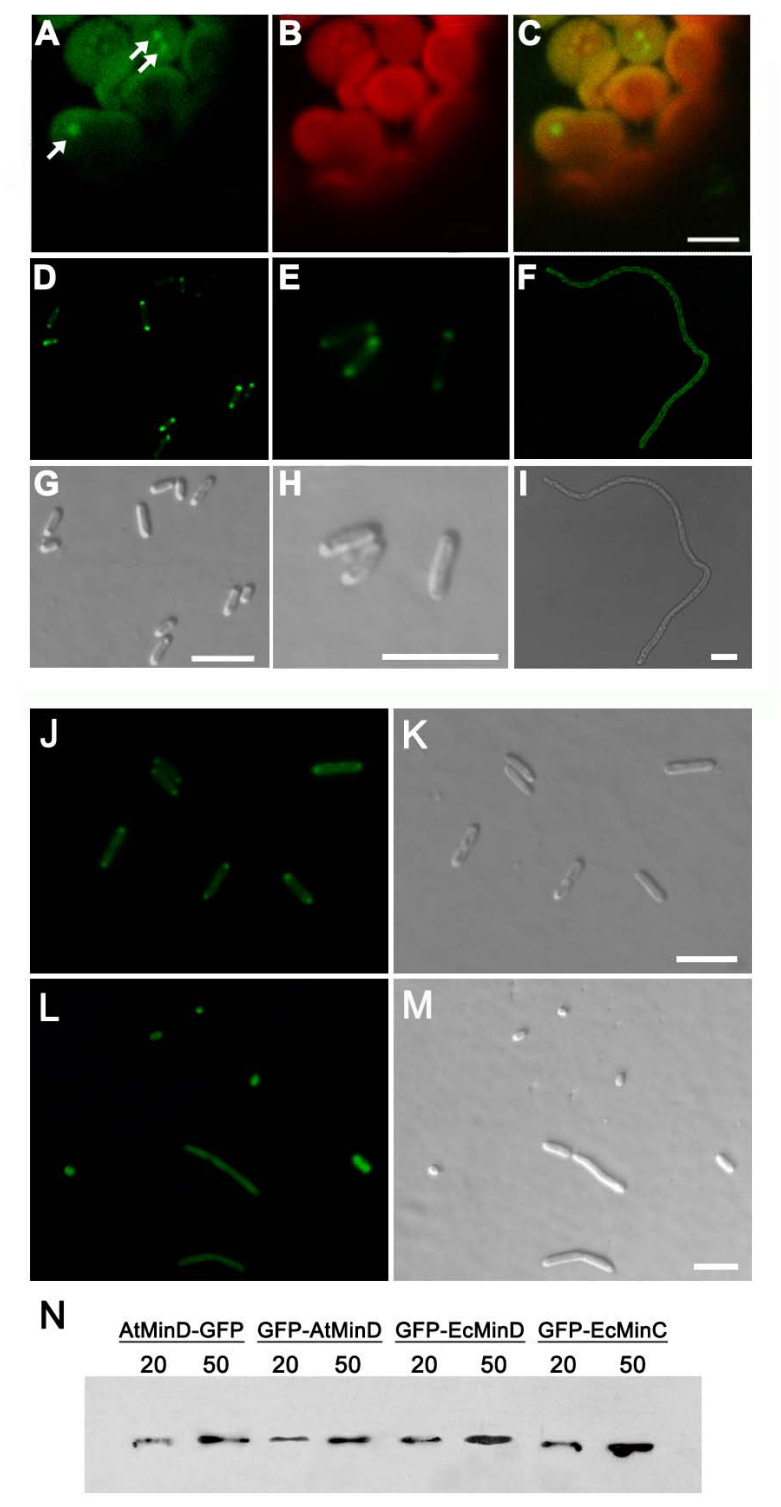
**Localization of AtMinD in *Arabidopsis *and *E. coli *with a GFP tag**. (A to C) AtMinD-GFP transiently expressed in an *Arabidopsis *protoplast. Arrows denote the localization of GFP in chloroplasts. (D and G) AtMinD-GFP expressed in *E. coli *HL1 mutant. (E and H), GFP-AtMinD expressed in *E. coli *HL1 mutant. (F and I) GFP-EcMinD expressed in *E. coli *HL1 mutant, (J and K) GFP-EcMinC and AtMinD expressed in *E. coli *RC1 mutant, (L and M) GFP-EcMinC expressed in *E. coli *RC1 mutant, (N) Immuno blot analysis. AtMinD-GFP, GFP-AtMinD and GFP-EcMinD were expressed in the HL1 mutant; GFP-EcMinC was expressed in the RC1 mutant with AtMinD. All the cells were grown with 20 or 50 μM IPTG. (A, D, E, F, J and L), GFP; (B), Chlorophyll; (C) Overlay; (G, H, I, K and M), DIC. Bars are 5 μm.

To understand the function of AtMinD in *E. coli*, AtMinD-GFP and GFP-AtMinD were expressed in HL1 mutant (Δ*MinDE*) (Figure 2D, E, G and 2H). Similar to AtMinD, AtMinD-GFP and GFP-AtMinD can complement the minicell phenotype of HL1 mutant (Δ*MinDE*) with 50 μM IPTG (Table 1 and Table 2). However, the complementation of the phenotype by AtMinD-GFP was not as good as the complementation by AtMinD (Table 1 and Table 2). This could be because the GFP tag partially affects the function of AtMinD-GFP. We have also tried to induce the expression of AtMinD-GFP with different concentration of IPTG (our unpublished results) and found that the mutant phenotype was complemented best with 50 μM IPTG, the same concentration as that for the complementation by AtMinD. This suggests that, although AtMinD-GFP may not be as effective as AtMinD for the complementation, both of them may interact with other division proteins with a similar stoichiometry and the interaction may not be affected by a GFP tag.

In the complemented mutant cells, AtMinD-GFP and GFP-AtMinD were localized to puncta at the polar regions of the cell (Figure 2D and 2E). With a chloroplast targeting transit peptide, AtMinD-GFP fusion protein transiently expressed in *Arabidopsis *protoplasts was localized to puncta in chloroplasts (Figure 2A, B and 2C). The green autoflorescence from chloroplasts wee dimmer than the signal from GFP (Figure 2A) and similar to that of untransformed cells (data not shown). This localization pattern is very similar to that of the AtMinD-GFP in stable transgenic *Arabidopsis *plants [19]. We have observed very carefully with time lapse images as people have done previously [22,23] for many cells with several repeats and never found the oscillation of AtMinD-GFP and GFP-AtMinD from one pole to another in the complemented *E. coli *HL1 mutant cells (Δ*MinDE*) or the chloroplasts in *Arabidopsis *(data not shown).

In *E. coli*, MinD is localized to the membrane and oscillates to one pole or another with a cytosolic protein MinC [8]. This oscillation is driven by MinE [8]. By oscillating in the cell and depolymerizing the FtsZ filaments at polar regions, the MinCD complex keeps the cell division apparatus at the midpoint of the cell [8]. Without the driver EcMinE, GFP-EcMinD was localized throughout the cell membrane with no oscillation and cells were long filaments (Figure 2F and 2I). This is probably due to a lack of FtsZ polymerization anywhere in the cell. However, a non-oscillating AtMinD can complement the phenotype of HL1 mutant (Figure 1E, Figure 2D and 2E and Table 1). In this complemented mutant, MinE is also absent and AtMinD is simply localized to the polar region of the cell. Therefore, *E. coli *can divide at the midpoint of the cell without an oscillating Min system.

So far we don't know why AtMinD is localized to the polar region in *E. coli *cells. Compared with chloroplasts, *E. coli *cells are much smaller and have a rod shape. By just localized to the polar region, AtMinD may keep the FtsZ ring and the division site at the midpoint of the cell. Since EcMinD depolymerize the FtsZ filaments at the non-division site through its interacting protein EcMinC [8], it is also likely that AtMinD interacts with and functions through EcMinC.

To test this prediction, GFP-EcMinC and AtMinD were coexpressed at 50 μM IPTG in RC1 mutant (Figure 2J and 2K). The mutant phenotype was rescued and GFP-EcMinC was localized to puncta at cell ends except that there was some signal in the cytosol. Without AtMinD, GFP-EcMinC was distributed evenly throughout the cell in RC1 mutant (Figure 2L and 2M). These data further suggest that AtMinD may interact with EcMinC and helps interpret the complementation of HL1 mutant by AtMinD.

To get an idea of the levels of GFP-AtMinD, GFP-EcMinD and other GFP fusion proteins, an immuno-blot was done (Figure 2N). The levels of these proteins were very close at the same concentration of IPTG. This is probably is because their coding genes are in similar vectors and under the control of the same promoter. The level of GFP-EcMinD probably was a little higher than that of GFP-AtMinD. This could be due to a better codon usage, higher stability etc. EcMinD rescues the mutant phenotype best at 20 μM IPTG, while AtMinD and its GFP fusion proteins rescues the mutant phenotype best at 50 μM IPTG. This probably is because their working mechanisms or (and) their activities are different.

### AtMinD interacts with EcMinC

**Figure 3:**
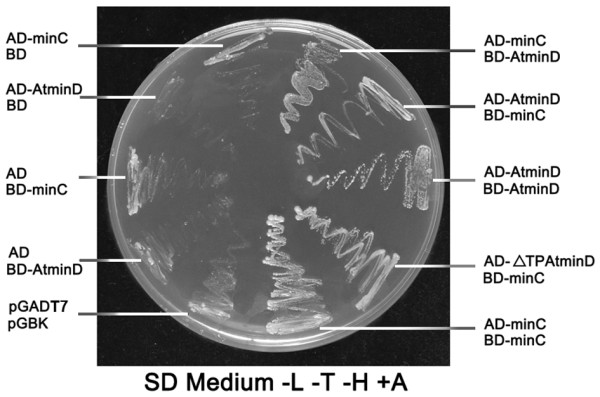
**Interactions of EcMinC and AtMinD examined by yeast two hybrid analysis**. Yeast cells grown without Leucine (L), Tryptophan (T) and Histidine (H), but with 3-AT. ΔTP, deletion of the chloroplast transit peptide. SD, synthetic defined.

To further explore the function of AtMinD, we studied the protein-protein interaction between AtMinD and EcMinC. First, we tested this by yeast two hybrid (Figure 3). In the yeast strain AH109 we used, certain genes for the biosynthesis of histidine, leucine and tryptophan are not expressed. If two proteins fused to the bait and prey respectively interact, the genes for the synthesis of histidine, leucine and tryptophan will be induced and the yeast cell will be able to grow without histidine, leucine and tryptophan. Because this system is leaky, 3-AT was used to reduce the basal level. As shown in Figure 3, full length AtMinD can interact with EcMinC no matter whether it is fused to the activation domain or the binding domain. The presence or the absence of the chloroplast transit peptide had no effect on the interaction between AtMinD and EcMinC (Figure 3). Both AtMinD and EcMinC can self-interact (Figure 3).

**Figure 4:**
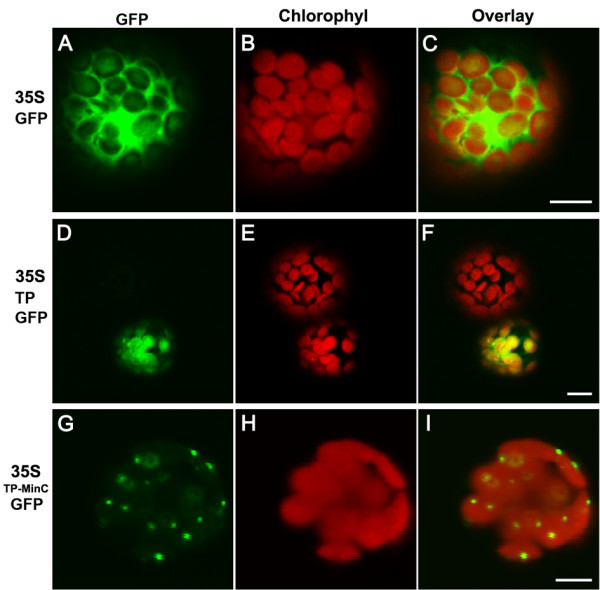
**Localization of a chloroplast-targeted EcMinC-GFP in *Arabidopsis***. (A to C) 35S-GFP transiently expressed in an *Arabidopsis *protoplast; (D to F) 35S-TP-GFP transiently expressed in *Arabidopsis *protoplasts; (G to I) 35S-TP-EcMinC-GFP transiently expressed in an *Arabidopsis *protoplast. All bars, 5 μm.

We also tested whether AtMinD can interact with EcMinC in chloroplasts. EcMinC fused with the N-terminal chloroplast transit peptide from Rubisco small subunit and a C-terminal GFP was transiently expressed in *Arabidopsis *protoplasts. Interestingly, EcMinC-GFP was localized to puncta in chloroplasts (Figure 4G, H and 4I), a pattern similar to that of AtMinD-GFP in chloroplasts [20,24]. This probably is because the endogenous AtMinD has a punctate localization pattern and it can interact with EcMinC-GFP. It has been shown that overexpression of chloroplast-targeted EcMinC in plants inhibits the division of chloroplasts [25]. In *E. coli*, EcMinC interacts with EcMinD to be associated with membrane and to inhibit FtsZ polymerization at the polar region [8]. These data suggest that EcMinC may interact with AtMinD in chloroplasts.

**Figure 5:**
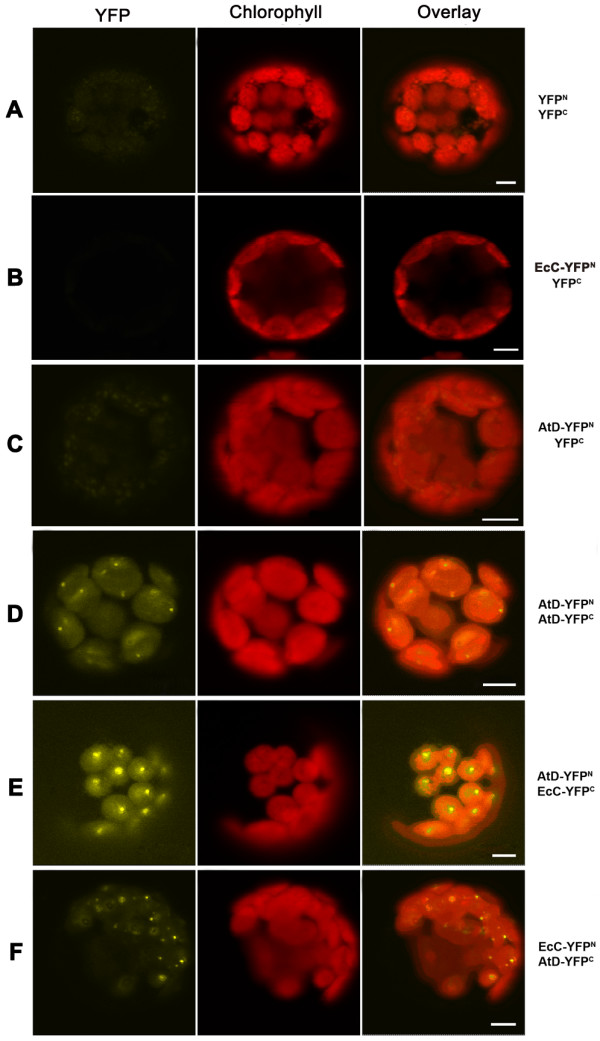
**Interactions of EcMinC and AtMinD examined by BiFC assay in *Arabidopsis *protoplasts**. (A) coexpression of 35S-YFP^N ^and 35S-YFP^C^; (B) 35S-TP-EcMinC-YFP^N ^and 35S-YFP^C^coexpression; (C) 35S-AtMinD-YFP^N ^and 35S-YFP^C^coexpression; (D) 35S-AtMinD-YFP^N ^and 35S-AtMinD-YFP^C^coexpression; (E) 35S-AtMinD-YFP^N ^and 35S-TP-EcMinC-YFP^C ^coexpression; (F) 35S-TP-EcMinC-YFP^N ^and 35S-AtMinD-YFP^C^coexpression. Bars, 5 μm.

To further confirm the interaction between AtMinD and EcMinC, we did a BiFC analysis based on the reconstitution of YFP fluorescence when nonfluorescent N-terminal YFP (YFP^N^) and C-terminal YFP (YFP^C^) fragments are brought together by two interacting proteins in living plant cells. These two proteins were fused with a chloroplast transit peptide and a part of YFP and transiently coexpressed in *Arabidopsis *protoplasts (Figure 5). AtMinD was tested by being fused with either YFP^N ^or YFP^C ^tag at the C-terminus for the interaction with EcMinC which has an YFP^C ^or YFP^N ^at the C-terminus (Figure 5E and 5F). In both cases, a strong YFP signal was detected at puncta in chloroplasts in contrast to the negative controls (Figure 5A, B and 5C). It has been shown that AtMinD can self interact by FRET analysis [20] and BiFC assay [26]. Here as a positive control, AtMinD self-interacts at puncta in chloroplasts by BiFC assay (Figure 5D). Overall, our data strongly suggest that AtMinD can interact with EcMinC.

It is interesting that AtMinD can still recognize EcMinC. However, no MinC homologue has been found in *Arabidopsis *and other higher plants yet. There are at least two possibilities. First, there are a lot of differences between chloroplasts and cyanobacteria in their structure, composition and function etc. The division apparatus of chloroplasts has evolved during evolution so that MinC might have been lost in higher plants and its function has been taken by another protein. Second, the sequence of MinC is less conserved than that of MinD in bacteria (data not shown). MinC could be too divergent to be recognized by sequence in higher plants.

It is hard to understand why AtMinD is localized to static puncta in chloroplasts in previous study [20] instead of a dynamic oscillating pattern. Here we show that AtMinD is localized to puncta at the polar regions in *E. coli *cells (Figure 2D and 2E) and puncta in chloroplasts (Figure 2A). By interacting with either endogenous or transiently expressed AtMinD, EcMinC-GFP, EcMinC-YFP^N ^and EcMinC-YFP^C ^are localized to puncta in chloroplasts too. These data further suggest that the punctate localization pattern of AtMinD in chloroplasts shown before [20,24] may be true. There are usually only one or two GFP-labeled puncta in one chloroplast. It is possible that chloroplasts constrict in-between puncta. However, this hasn't been confirmed.

So far, it seems that the working mechanism of Min system in plastids is a lot different from that in *E. coli*. However, the study of Min system in plastids is limited and our understanding about it is not very clear. AtMinE seems to have an antagonistic role to AtMinD in plastid, because the chloroplast division phenotype caused by overexpression of AtMinE was similar to that caused by antisense suppression of AtMinD in *Arabidopsis *[17,19]. This kind of relationship is still similar to that of EcMinE and EcMinD [7]. Further study needs to be done to understand the working mechanism of AtMinE in plastids.

## Conclusion

In this paper, we have shown that AtMinD was localized to puncta at the polar region and is functional in *E. coli*. AtMinD may function through the interaction with EcMinC. It is not necessary for AtMinD to oscillate to keep the cell division site at the center of *E. coli *cells. In *Bacillus subtilis*, the MinCD proteins are localized to polar regions without oscillation [27]. There is no MinE in *B. subtilis *[27]. Instead, another protein DivIVA tethers MinCD to poles of the cell and prevents FtsZ polymerization and division apparatus assembly at the end of the cells [27]. AtMinD and EcMinC in *E. coli *HL1 mutant (Δ*MinDE*) may work in a manner similar to the BsMinD and BsMinC in *Bacillus subtilis*.

## Methods

### *E. coli *strains and bacterial expression vector construction

The *E. coli *strains used in this study were DH5α, HL1 (Δ*MinDE*) [21] and RC1 (Δ*MinCDE*) [28]. The culture were grown to OD600 = 0.4 – 0.45 at 37°C in LB medium with 100 μg/ml ampicillin, 50 μg/ml kanamycin or 25 μg/ml chloramphenicol respectively as required. *AtMinD *lacking the coding region of the N-terminal 57 amino acid residues were amplified by using primers: AD1F1, CGGAATTCAACAAGGAATTTCTATGCCGGAACTCGCCGGAGAAACGC and AD1R1, GCAAGCTTTTAGCCGCCAAAGAAAGAGAAGA. *EcMinD *and *EcMinDE *were amplified from the genomic DNA of DH5α by primers: EcDF1, GCGGAATTCAAGGAATTTCTATGGCACG and EcDR1, GCGAAGCTTATCCTCCGAACAAGCG or EcER1, GCGAAGCTTA CAGCGGGCTTATTTCAG. These PCR products were cloned into *pMLB1113 *[1] between the EcoRI and HindIII restriction enzyme cutting sites to generate *pM1113-AtMinD*, *pM1113-EcMinD *and *pM1113-EcMinDE*. To obtain an AtMinD-GFP expression vector in *E. coli*, the *AtMinD *gene was first amplified with primers: AD1F2, CGGGATCCCATGCCGCGTATCGTCGTTATC and AD1R2, CATACCATGGTGCCGCCAAAGAAAGAGAAGA and inserted into *pEGFP *(Clontech, CA) between the BamHI and NcoI restriction enzyme cutting sites. Then the *AtMinD-GFP *fusion gene was PCR-amplified with primers AD1F1 and GFPR, CCGAAGCTTTTACTTGTACAGCTCGTC and introduced into vector *pMLB1113 *between the EcoRI and HindIII restriction enzyme cutting sites. To obtain GFP-AtMinD and GFP-EcMinD expression vectors, *GFP *was amplified from *pEGFP *plasmid by primers CGAATTCAACAAGGAATTTCTATGGTGAGCAAGGGC/GCTCTAGACTTGTACAGCTCGTC and cut by EcoRI and XbaI. *AtMinD *or *EcMinD *were PCR amplified by primers AD1F3, GCTCTAGAATGCCGGAACTCGCCGGAGAAACGC/AD1R1 or EcDF2, GCTCTAGAATGGCACGCATTATTGTTGT/EcDR1 and cut by XbaI and HindIII. *GFP *and *AtMinD *or *EcMinD *were ligated together *in vitro *and then inserted into *pMLB1113 *between EcoRI and HindIII cutting sites. For the construction of GFP-EcMinC expression vectors, *EcMinC *was amplified by MCF1, GCTCTAGAATGTCAAACACGCCAATCG and MCR1, ATGGATCCTCAATTTAACGGTTGAACGG and cut by XbaI and BamHI. *EcMinC *and the *GFP *gene above were ligated together *in vitro *and then inserted into *pMLB1113 *between EcoRI and BamHI cutting sites. To express AtMinD and GFP-EcMinC together, *AtMinD *was amplified by AD1F4, CGGGATCCAACAAGGAATTTCTATGCCGCGTATCGTCGTTATC and AD1R1, cut by BamHI and HindIII and then inserted into *pMLB1113-GFP-EcMinC*. All the constructs above were transformed into HL1 mutant (Δ*MinDE*) or RC1 mutant (Δ*MinCDE*) respectively.

### Yeast two-hybrid analysis

*AtMinD *and Δ*TPAtMinD *were PCR-amplified with primers YDF1, GGGTTTCATATGGCGTCTCTGAGATTGTTC and YDR, CGGGATCCTTAGC CGCCAAAGAAAG or YDF2, GGGTTTCATATGCCGGAACTCGCCGGAGA AACGC and YDR, cloned into pGADT7 and pGBK (Clontech, CA, USA) which were cut by NdeI and BamHI. EcMinC was amplified with primers CF, CGGAATTCATGTCAAACACGCCAATCG and CR, ATGGATCC TCAATTTAACGGTTGAACGG, then introduced into pGADT7 and pGBK between the restriction enzyme cutting sites EcoRI and BamHI. All the constructs were first made in *E. coli *DH5α and then transformed into yeast strain AH109 by using the lithium acetate method. If the two proteins fused to the bait and prey respectively can interact with each other, the cotransformed yeast cells will grow in the absence of leucine, tryptophan and histidine and in the presence of 3 mM 3-AT [29-31], according to the protocol from Clontech.

### AtMinD and EcMinC localization and BiFC assay in Arabidopsis

Complete open reading frame of *AtMinD *was amplified with gene-specific primers GCTCTAGAATGGCGTCTCTGAGATTGTTC and GCCTCGAGGCCGCCAAAGAAAGAGAAGA to remove the stop codon and have a C-terminal in-frame fusion with the coding region of GFP, YFP^N ^(1–158 amino acid residues of YFP) and YFP^C ^(159–239 amino acid residues of YFP). The PCR product was cut by XbaI and XhoI, and cloned into *PUC19-35S-MCS-GFP*, *PUC19-35S-MCS-YFP*^*N *^and *PUC19-35S-MCS-YFP*^*C *^which were constructed as previously described [32,33]. These gene manipulations generated *PUC19-35S-AtMinD-GFP*, *PUC19-35S-AtMinD-YFP*^*N *^and *PUC19-35S-AtMinD-YFP*^*C*^.

To obtain appropriate localization of EcMinC which had no chloroplast transit peptide, we used the first 58 amino acid residues from the Rubisco small subunit (At5g38410) in *Arabidopsis thaliana*. The coding region was amplified with primers TPF, GCTCTAGAGTAATGGCTTCCTCTATGCTC and TPR, GCGGATCCCTTCATGCAGCTAACTCTTCC, cloned into *PUC19-35S-MCS-GFP *between XbaI and BamHI cutting sites to obtain *PUC19-35S-TP-GFP*. *EcMinC *(GeneBank J03153) was PCR-amplified with primers MinCF, GCGGATCCATGTCAAACACGC CAATCG and MinCR, GCCTCGAGATTTAACGGTTGAACGGTCAAAG and cut by BamHI and XhoI and cloned into the above vector to generate *PUC19-35S-TP-MinC-GFP*.

The *GFP *gene in *PUC19-35S-TP-MinC-GFP *was replace with YFP^N ^and YFP^C ^to generate *PUC19-35S-TP-MinC-YFP*^*N *^and *PUC19-35S-TP-MinC-YFP*^*C*^.

For the localization and BiFC protein interaction analysis of AtMinD and EcMinC, the above constructs were transformed or cotransformed into *Arabidopsis *protoplasts by PEG-mediated method [34].

### Microscopy and phenotype analysis

Differential interference contrast (DIC) microscopy and fluorescence microscopy were done by using Leica multifunctional microscope. The fluorescence in *Arabidopsis *protoplasts was detected by using Leica confocal laser scanning Microscope SP2. Images were processed with PHOTOSHOP software (Adobe Systems, San Jose, CA, USA).

*E. coli *cells in exponential growth stage and with optical density (600 nm) values between 0.4 and 0.45 were collected by centrifugation at 13 000 g for 15 minutes and the pellets were resuspended in 0.05% low melting point agar to eliminate the uneven distribution of cells on microscope slides. AxioVision AC software (Zeiss, Germany) was used to measure the size of cells. Approximately 200 cells were measured each time and three or four repeats were done. SigmaPlot 9.0 (SYSTAT Statistics, CA, USA) was used for statistical analysis of the phenotype. To score visible cell constriction sites, more than one hundred septa were counted for each genotype. Septa which were misplaced at or near a cell pole were regarded as polar septa. The percentage of polar septa for each genotype was calculated to reflect the cell division phenotype.

### Immuno-blot analysis

*E. coli *cells were broken by ultrasonication in the extraction buffer (50 mM Tris HCl pH 8.0, 25 mM NaCl, 2 mM EDTA) and the crude total protein concentration was determined with a Dc protein assay kit (Bio-Rad). 5 μg of proteins were applied to each lane for SDS-PAGE. Immuno-blot analysis was done with polyclonal anti-GFP antibodies (Sigma, G1544).

## Abbreviations

At: *Arabidopsis thaliana*; Ec: *Escherichia coli*; Bs: *Bacillus subtilis*; BiFC: bimolecular fluorescence complementation; GFP: green fluorescent protein; YFP: yellow fluorescent protein; 3-AT: 3-aminotriazole.

## Authors' contributions

YH, HG, MZ and YH designed the experiments. MZ and JJ carried out the experiments. HG, YH, and MZ analyzed the data and wrote the paper. All authors read and approved the final manuscript.
